# Transcaval Access to the Abdominal Aorta: indications of Interest to Surgeons and a Comprehensive Literature Review

**DOI:** 10.21470/1678-9741-2019-0240

**Published:** 2020

**Authors:** Francisco S. Lozano Sánchez, Ignacio Cruz González, Roberto Salvador Calvo, Pedro Luis Sánchez Fernández

**Affiliations:** 1 Department of Angiology and Vascular Surgery, Hospital Universitario de Salamanca, Salamanca, Spain.; 2 Department of Cardiology, Hospital Universitario de Salamanca, Salamanca, Spain.

**Keywords:** Endoleak, Transcatheter Aortic Valve Replacement, Aortic Aneurysm, Abdominal, Blood Vessel Prosthesis Implantation, Endovascular Procedures, Aorta, Surgeons

## Abstract

We performed a review of the literature (until August 01, 2019) on the occasion of the first transcaval approach for transcatheter aortic valve implantation in our hospital. This review focuses mainly on the indications of this alternative access route to the aorta. It may be useful for vascular surgeons in selected cases, such as the treatment of endoleaks after endovascular aneurysm repair and thoracic endovascular aneurysm repair. We describe historical aspects of transcaval access to the aorta, experimental studies, available case series and outcomes. Finally, we summarize the most significant technical aspects of this little-known access.

**Table:** 

Abbreviations, acronyms & symbols	
**ChEVAR** **EVAR** **TAVI** **TAVR** **TC** **TEVAR**	**= Chimney endovascular aneurysm repair** **= Endovascular aneurysm repair** **= Transcatheter aortic valve implantation** **= Transcatheter aortic valve replacement** **= Transcaval** **= Thoracic endovascular aneurysms repair**

## INTRODUCTION

Percutaneous aortic valve replacement, also known as transcatheter aortic valve implantation (TAVI) or transcatheter aortic valve replacement (TAVR), was first performed by Alan Cribier in 2002 (Rouen, France)^[1]^. In Spain, the first implantation was performed in 2007, followed shortly by the first in our hospital. This procedure is currently performed in many hospitals. Collaboration between the Department of Angiology and Vascular Surgery and the Department of Cardiology in our hospital includes providing, in selected cases, an alternative to conventional vascular access approach (percutaneous femoral artery) for TAVI^[2]^.

In large series, between 12 and 19% of patients are not candidates for conventional femoral access^[3,4]^; however, the current frequency of access to non-femoral arteries is lower. Thus, alternative accesses have been described: transthoracic (transapical or transaortic) and extrathoracic (trans-subclavian and transaxillary, transcarotid, transcaval) accesses. The experience with these access routes has shown their advantages and disadvantages^[5]^.

In our hospital, a transcaval (TC) TAVI procedure was performed (October 7, 2018) with favorable results. It was the first TC approach for TAVI in our hospital and the second in Spain. The aim of this report is to review the indications for this vascular approach (technical details, historical aspects, preclinical and clinical outcomes), which may be useful for vascular surgeons.

## METHODS

We carried out a review of the literature. We found 218 references in the PubMed/MEDLINE database (August 1, 2019) using the following keywords: transcaval (142 references), caval-aortic (15 references), transcaval aortic access (40 references), transcaval and endoleak (19 references), and transcaval and TEVAR (2 references). Among the 218 references, those that appeared simultaneously for two or more keywords were removed; subsequently, after reading the titles or abstracts, references not related to this subject were also excluded. In addition, references related to this subject but very narrative and repetitive were removed. Finally, 25 reports were selected: three on the access route technique^[6-8]^, one experimental study^[9]^; three cohort references (one retrospective and two prospective) for TAVI^[10-12]^; two cases on TC access for TAVI through a conventional aortic graft^[13,14]^; one case on TC access for TAVI through partially thrombosed infrarenal aortic aneurysm^[15]^; one case on TC access for TAVI in a patient with duplicated inferior vena cava^[16]^; one case on TC access for Biventricular Impella placement^[17]^; eight references on six cohorts (retrospective) on the treatment of type II endoleaks after EVAR^[18-25]^; two cases on treatment of type I endoleaks after EVAR and after chimney endovascular aneurysm repair (ChEVAR) respectively^[26,27]^; two cases on thoracic endovascular aneurysms repair (TEVAR)^[28,29]^; and one systematic review on the transcaval access to the aorta^[30]^.

### Technical Aspects of Transcaval Access to the Aorta

**Fig. 1:**
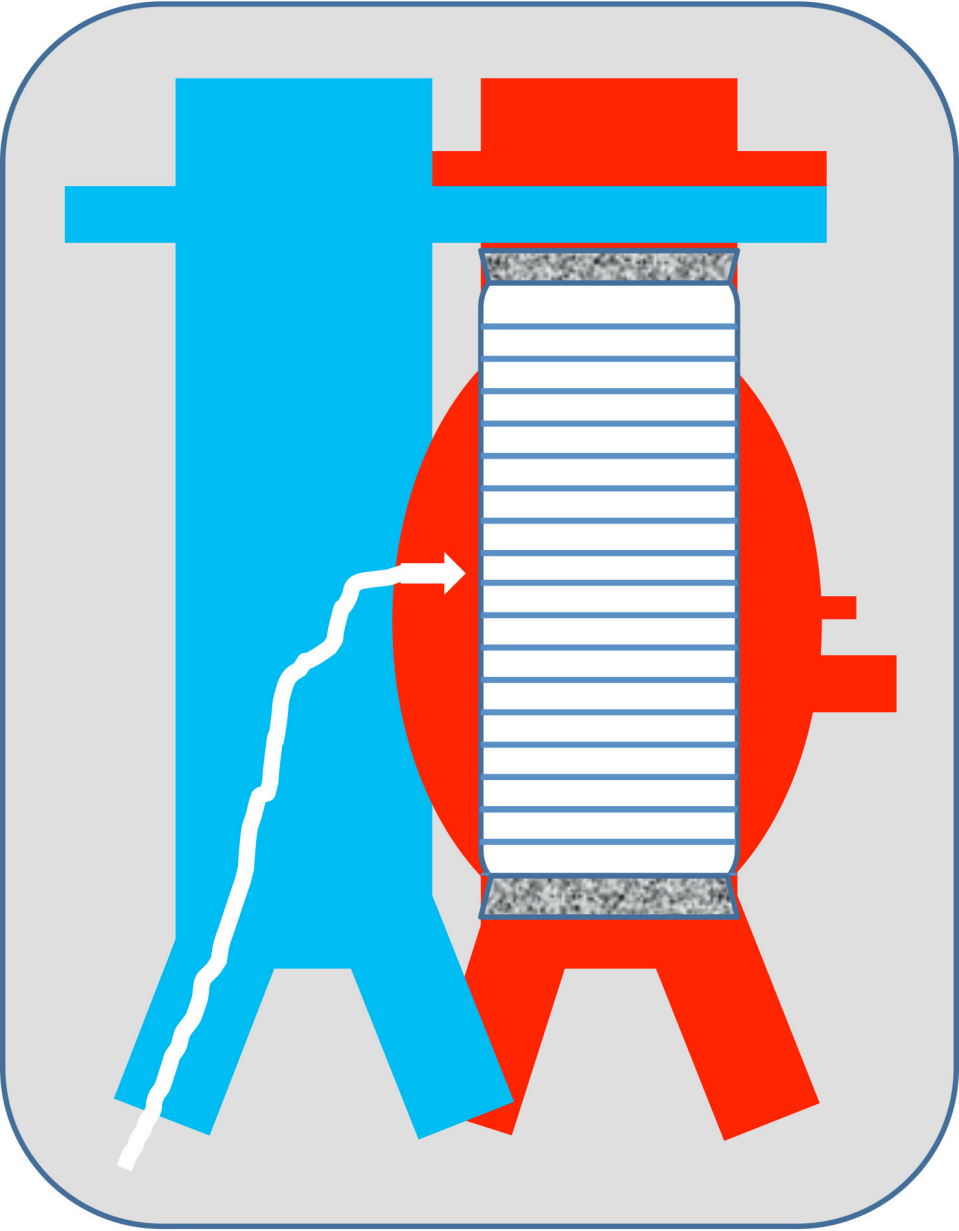
Scheme of transcaval access approach to the aneurysmal sac for embolization of type II endoleaks.

**Fig. 2:**
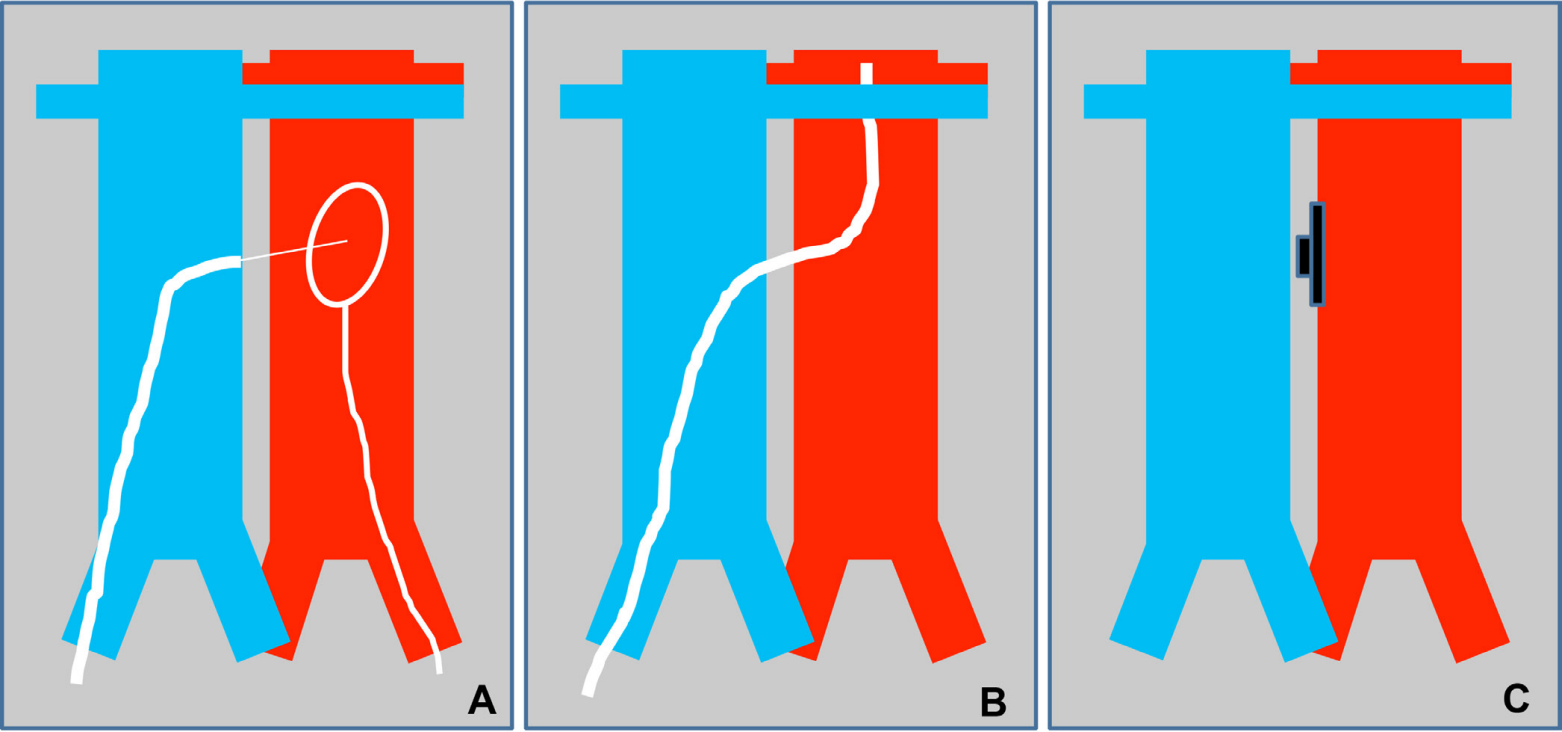
Scheme (main steps) of transcaval access to the aorta: A) access (transfemoral electrified guidewire), B) crossing (caval-aortic catheter), and C) closure of caval-aortic access.

**Fig. 3:**
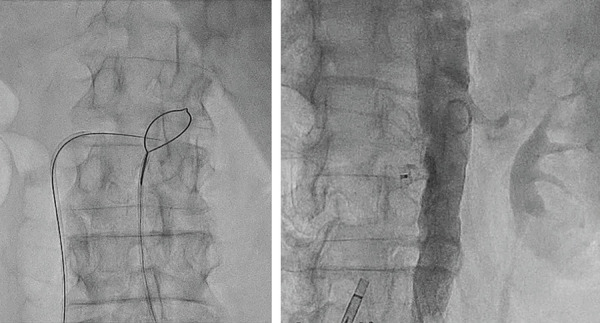
Real case (Hospital Universitario de Salamanca, October 7, 2018).

*There are two techniques*: 1) TC access to the aneurysm sac after EVAR, used for endoleaks (Figure 1); 2) TC access to the aorta, used for TAVI, Impella and TEVAR (Figures 2 and 3). The main difference between these techniques is the aortocaval fistula: it is larger in the second access route, since the introducer caliber is also larger. The most significant steps of the second technique, which is the most complex, are explained below^[6]^. 

*Planning*: A thoracic and abdominal CT scan should be performed to assess the subclavian, aortic, iliac and femoral arteries, to establish the relationship between the vena cava and the aorta, and to determine the most appropriate site for the vena cava puncture. A calcium-free region in the aorta without interposed structures, such as the bowel, should be chosen^[7]^. There should be sufficient distance from the renal artery and vein, the mesenteric vessels and the aortoiliac bifurcation to allow a cover stent to be implanted when the resulting aortocaval fistula is not closed. 

*Procedure*: Percutaneous femoral venous and arterial access. With a prepositioned catheter, simultaneous injections of contrast medium should be performed into the inferior vena cava and the aorta to mark the puncture site. Once marked, a snare loop should be placed in the aorta. The snare should be aligned with the catheter, previously positioned in the inferior vena cava in two projections. In this way, the guidewire advances towards the aortic snare and it is captured by the snare. A rigid one-piece non-hydrophilic guidewire should be introduced. The proximal end is connected to an electrosurgery pencil that allows advancement across the structures. Once confirmed the intraluminal guidewire position, the electrosurgery pencil should be removed and the guidewire snared and advanced towards the aortic arch. A 2.5 to 3.00 mm balloon can be used for dilatation of the fistulous tract. It allows the exchange for a high support guidewire. We used a microcatheter over the guidewire that allowed the exchange to a support guidewire; we did not used the balloon for dilatation. The percutaneous introducer sheath (18 Fr) should be advanced through the guide. Once the distal end of the introducer was advanced into the abdominal aorta, valve implantation can be performed in the same way as the conventional femoral arterial access. After valve implantation, the aortocaval shunt should be closed using either an Amplatzer-type duct occluder (first generation) or the new, purpose-built occluder that is currently being trialed by Transmural Systems. Contrast medium was injected to exclude retroperitoneal bleeding. In case of bleeding, it is advisable to inflate a compliant balloon, used for vascular graft remodeling. If bleeding persists, a coated aortic stent is advised. The introducers may be removed after confirming the absence of bleeding (Figures 2 and 3). The above-described procedure can be visualized in the publication of Muhammad and Tokarchik^[8]^, who reported that TC procedures have been performed in approximately 450 cases worldwide. This proves the safety and effectiveness of the TC approach. Patients were usually discharged 48 hours after the procedure. They should be followed-up with CT angiography at one month and one year to rule out aortocaval fistula, pseudoaneurysm and other vascular complications. However, our knowledge of the excellent results after 1 year explains why most practitioners now omit the 12-month routine follow-up CT scan, except in situations where the patient's symptoms indicate that this would be worthwhile^[12]^.

### Historical Aspects of Transcaval Access

The TC access is mainly due to Robert J. Lederman (Cardiovascular and Pulmonary Branch, National Heart Lung and Blood Institute, National Institutes of Health, Bethesda, Maryland) and Adam B. Greenbaum (Division of Cardiology, Henry Ford Health System, Detroit, Michigan) and their colleagues, who have worked hard on TC access as a route for TAVI.

The initial human experience started after an experimental study in swine with favorable outcomes^[9]^. The series by Greenbaum et al.^[10]^ demonstrated the viability of this access route as an alternative to femoral puncture for TAVI in 19 patients. A few years later, Greenbaum et al.^[11]^ published a comprehensive observational prospective study in 100 patients who underwent TC TAVI.

However, there are other precedents. The TC access to the aorta was first used accidentally during a translumbar embolization of type II endoleaks after endovascular aneurysm repair (EVAR)^18]^. However, the access route used by Stavropoulos should be called right-sided translumbar transcaval approach. Thus, the real first TC access should be attributed to Mansueto et al.^[19,20]^.

The TC access to the aorta has several potential indications within our specialty (angiology and vascular surgery). In addition to embolization of type II endoleaks^[18-25]^, the treatment of type I endoleaks^[26,27]^ was developed in 2015. In 2015 and 2017, two cases of TC access for thoracic endovascular aortic repair (TEVAR)^[28,29]^ were published. The aim of this review is to describe the current status of these indications and some technical details.

### Experimental Studies: Advantages and Disadvantages of Transcaval Access

Halabi et al.^[9]^ published a study on 14 swine reporting the advantages and disadvantages of caval-aortic access. First, femoral veins are more compliant than arteries to allow wider cannulae. Second, large arteriovenous fistulas, such as aortocaval fistulas, are pathological, but they are not immediately life-threatening. Conversely, these fistulas avoid hemorrhage by decompressing high-flow arterial ruptures in the venous system. Third, the inferior vena cava abuts the infrarenal aorta without interposed critical structures. Fourth, a marketed device was used to occlude an arteriovenous communication. The radiofrequency energy application through a guidewire was inspired by transseptal puncture facilitated by an atrial transseptal needle connected to an electrosurgery generator.

Limitations included differences between human and swine. Patients may be susceptible to major complications such as aortic dissection, aortic thrombosis, retroperitoneal hemorrhage, vena cava thrombosis and venous thromboembolism, lymphatic injury, or device migration or embolization.

The authors of this experimental study (from Dr. Lederman’s above-mentioned group) concluded that large therapeutic devices could be introduced into the aorta via the inferior vena cava through a simple catheter procedure, and that the fistula could be uneventfully closed despite the anticoagulation used during the procedure. The intentional failure of closing the caval-aortic access tract is well-tolerated and can be easily corrected. These experimental findings justify human testing.

### Physiology of Transcaval Access To the Abdominal Aorta

As McCabe ingeniously mentions in his editorial “Traversing the Chasm”^[31]^, TC access disrupts all our concepts of vascular access because, as he says: “prior to knowing this access, would someone make a hole between the vena cava and the aorta? Surely not, because we would all think that this would lead to a retroperitoneal hemorrhage or an aortocaval fistula with consequences, even catastrophic ones”.

The fact is that this apparently aggressive technique behaves in a banal way and, as the results at 1 and 12 months show^[12]^, its practice is possible. The technique is effective (it works) and safe because it does not induce severe retroperitoneal hemorrhage and its closure is safe because it does not cause aortocaval fistula. This avoids the consequences well known to vascular surgeons, in which abdominal aortic aneurysms rupture spontaneously in the inferior vena cava^[32]^.

### Clinical Studies: Indications and Results

Some endovascular aortic procedures (TAVI, EVAR, TEVAR, ChEVAR, and others) require large caliber introducers. The small caliber or disease (tortuous, calcified, stenosed or obstructed arteries) of the iliac arteries prevents femoral artery access in a number of candidates that may be significant^[3-5]^. It is then necessary to look for alternatives, such as TC access. Table 1 shows the overall experience with TC access.

**Table 1:** Clinical experience with transcaval access to the aorta.

Author/s [reference]	Journal, year (authors' country)	No. of cases	Technical success (%)	Follow-up (months)
**TAVI**				
- Greenbaum et al.^[10]^	J Am Coll Cardiol, 2014 (US)	19	89.5	111±57
- Greenbaum et al.^[11]^	J Am Coll Cardiol, 2017 (US)	100	99	1
- Lederman et al.^[12]^	J Am Coll Cardiol Interv, 2019 (US)	100	100	12
TAVI*				
- Lederman et al.^[13]^	Catheter Cardiovasc Interv, 2015 (US)	1	100	1
- Lanz et al.^[14]^	Can J Cardiol, 2018 (Switzerland)	1	100	6
- Piayda et al.^[15]^	Eur Heart J, 2019 (Germany)	1	100	1
- Fanari et al.^[16]^	Cardiovasc Revasc Med, 2017 (US)	1	100	1
**Biventricular Impella**				
- Kamioka et al.^[17]^	Catheter Cardiovasc Interv, 2019 (US)	1	100	5
**Type II endoleaks**				
- Stavropoulos et al.^[18]^	J Vasc Interv Radiol, 2003 (US)	9	100	12
- Mansueto et al.^[19]^	Cardiovasc Intervent Radiol, 2005 (Italy)	3	100	1
- Mansueto et al.^[20]^	J Vasc Surg, 2007 (Italy)	12	92	6
- Scali et al.^[21]^	J Vasc Surg, 2013 (US)	6	100	10.0±5.8
- Gandini et al.^[22]^	J Endovasc Ther, 2014 (Italy)	26	100	24.0±6.4
- Giles et al.^[23]^	J Vasc Surg, 2015 (US)	29	89.7	16.5±10.4
- Burley et al.^[24]^	J Vasc Surg, 2019 (US)	10	90	6
- Hyatt et al.^[25]^	CVIR Endovasc, 2019 (US)	1	100	1
**Type I endoleaks**				
- Gandini et al.^[26]^	J Endovasc Ther, 2015 (Italy)	1	100	12
- Massimi et al.^[27]^	J Vasc Surg, 2017 (US)	1	100	1
**TEVAR**				
- Uflacker et al.^[28]^	J Vasc Interv Radiol, 2015 (US)	1	100	1
- Fanari et al.^[29]^	Catheter Cardiovasc Interv, 2017 (US)	1	100	1

TAVI=transcatheter aortic valve implantation; TEVAR=thoracic endovascular aneurysm repair.

* The access was not directly to the aorta, but to a Dacron graft replacing the aorta due to previous aortic surgery^[13,14]^; through partially thrombosed infrarenal aortic aneurysm^[15]^; in a patient with duplicated inferior vena cava^[16]^.

After a preliminary study in 19 patients^[10]^, Greenbaum et al.^[11]^ published a comprehensive prospective observational study in 100 patients who underwent TC access for TAVI. TC access was successful in 99% of patients; closure (Amplatzer) was successful in 98% (one case required a coated stent). Inpatient survival was 96%, and 30-day survival was 92%. Major vascular complications related to TC access were 13%. Median length of stay was four days. Therefore, we may conclude that TC access for TAVI is a realistic alternative for patients ineligible for surgery (comorbidities and high risk) and without good options of conventional or alternative vascular access. The most feared complication related to this procedure is retroperitoneal hemorrhage, which may occur in 1-2%. Finally, a recent prospective multicenter study presented results from 12 months after access. Clinical follow-up, laboratory work and CT scans revealed no major vascular complications or clinical events (hemorrhages, fistulas, etc.) related to access^[12]^. Other than this and some other publications of such experiences, we are not aware of any reports of aortic or venous thrombosis, lymphatic injury, visceral lesion, or device migration.

A particular feature of the TC access is the possibility of performing the access through a previously implanted conventional aortic graft due to aortic pathology. This procedure has been successfully performed in two cases^[13,14]^. The first one had a polyester aortic graft implanted 15 years earlier for a type IV thoracoabdominal aneurysm^[13]^. The second patient had a bifurcated Dacron graft^[14]^. 

Other atypical situations are: TC access in a patient with abdominal aortic aneurysm or a double inferior vena cava, respectively^[15,16]^.

Finally, this access has also been successfully used to implant a Biventricular Impella in a patient without the possibility of other access^[17]^.

### Indications of Interest for Vascular Surgeons

#### Transcaval approach for embolization of endoleaks after EVAR

EVAR and TEVAR procedures were performed almost simultaneously for the first time by Volodos et al.^[33]^ and Parodi et al.^[34]^, whose fascinating story is beyond the scope of this review^[35,36]^. One of the main complications of these procedures are the endoleaks.

The term endoleak was first proposed by White et al.^[37]^ to define the incomplete exclusion of the blood flow from the aneurysm sac after endovascular repair by an endovascular graft. Endoleaks were categorized^[38]^ for a better management based on the origin of the blood flow (four types) and endotension (type V). 

Types I and II are of interest for this review:

Type I endoleaks: presence of perigraft blood flow caused by an inadequate seal at the proximal (IA) or distal (IB) end of a stent graft. They must be detected early, since they require immediate repair.

Type II endoleaks: these are the most common endoleaks. They occur from the retrograde blood flow of the collateral arteries excluded by the stent graft, typically from an inferior mesenteric artery (IIA), a lumbar artery (IIB) or others (e.g., the median sacral artery). The treatment remains controversial. In many cases, they resolve spontaneously. It can be treated in case of persistent flow and enlargement of the aneurysm sac (pressurization). There are multiple options for its management: transarterial embolization, direct injection of thrombogenic material into the aneurysm sac (CT-guided), selective arterial injection via lumbar puncture (CT-guided), laparoscopic branch ligation, open surgery, etc.

Injection of thrombogenic material into the aneurysm sac is usually performed by translumbar puncture. TC access to the aneurysm sac is an alternative access route.

There are several references to type II endoleaks in the literature^[18-25]^. Seven groups, five Americans and two Italians, have published their results in 93 patients; the results were successful in 90-100% (follow-up at 1-24 months) (Table 1). Access to the inferior vena cava is usually performed through the femoral vein; it has been performed via the right jugular vein only in some cases by Mansueto. Injection of thrombogenic material (thrombin, coils, etc.) into the aneurism sac is the standard technique in all series. Mortality was not reported in any series and morbidity is low (0-8%). However, recurrence varies in the different series (8-34%). This can be explained by the follow-up period (Table 2).

**Table 2:** Summary of type II endoleaks after EVAR cases repaired by transcaval embolization.

Author/s [reference]	No.	Age	Men	Vein access	Embolization technique	Mortality (30 days)	Complications (30 days)	Recurrence
Stavropoulos et al.^[18]^	9	-	-	-	-	-	-	-
Mansueto et al.^[19,20]^	12	79.0±5.3	92%	F(42%)/J (58%)	Standard	0	8%	8.3%
Scali et al.^[21]^	6	72.7±10.8	100%	Femoral	Standard	0	0%	34%
Gandini et al.^[22]^	26	75±5.6	-	Femoral	Standard	0	7.7%	15.4%
Giles et al.^[23]^	29	78±7.1	83%	Femoral	Standard	0	0%	17%
Burley et al.^[24]^.	10	82.0±7	80%	Femoral	Standard	0	0%	10%
Hyatt et al.^[25]^	1	75	100%	Femoral	Standard	0	0%	-

F=femoral; J=jugular

There are only two publications^[26,27]^ on type I endoleaks. Each publication reports a case. The most significant data and results are shown in Tables 1 and 3.

**Table 3:** Summary of type I endoleaks after EVAR cases repaired by transcaval embolization.

Author/s [reference]	No.	Age/sex	Repair indication	Previous surgery	Endograft	Material of embolization
Gandini et al.^[26]^	1	82/man	Type IA endoleak	EVAR (7 months)	Ovation	Coils and thrombin
Massimi et al.^[27]^	1	77/man	Gutter-related type I endoleak	ChEVAR* (1 month)	Not reported	Coils

EVAR=endovascular aneurysm repair; ChEVAR=chimney endovascular aneurysm repair,

* Superior mesenteric artery and two renal arteries.

#### Transcaval Access for TEVAR

TEVAR is usually performed via the femoral arteries. As an alternative to a case of unsuitable iliofemoral arterial approach, Uflacker et al.^[28]^ (from the group of Lederman, who described TC access) performed a TC access for TEVAR. The report was published in 2015, just one year after the description of this approach in humans. The procedure was successful; a Valiant stent graft (Medtronic^®^) was implanted and the aortocaval fistula was closed with an Amplatzer (St. Jude^®^).

Two years later, Fanari et al.^[29]^ performed a procedure with similar technique and results (Table 4). However, the patient developed a small type IA endoleak, which resolved spontaneously within a week. 

**Table 4:** Summary of thoracic aortic aneurysm (TAA) cases repaired by transcaval (TC) TEVAR.

Author/s [reference]	No.	Age/sex	Repair indication	Open surgery rejected	Indication TC	Endograft	Fistula closure
Uflacker et al.^[28]^	1	61/man	TAA 4.5-5.9 cm	High comorbidity	Iliac arteries <6 mm	Valiant (Medtronic^®^)	Amplatzer (St. Jude^®^)
Fanari et al.^[29]^	1	65/man	TAA 5.3-6.3 cm	High comorbidity	Unsuitable iliac access	Valiant (Medtronic^®^)	Amplatzer (St. Jude^®^)

Follow-up was very short in both cases (one month). The patients did not require a new intervention during this period. We believe that the most significant input of these two cases was their success. The future will show whether this technique can be performed more frequently. 

Finally, the systematic review by Wee et al.^[30]^ summarized the current status of this subject: "TC approach for endovascular aortic interventions for patients unsuitable for traditional access routes". The authors described the outcome of 209 patients (TAVI, type II endoleaks and TEVAR) with a mean age of 79.5±5.1 years and 51.2% men. The overall technical success rate was 96.2%, with a mortality rate of 4.3% and a mean follow-up of 17.9±19.8 months.

## CONCLUSION

In conclusion, our review shows potential indications for an alternative aortic access route. This may be useful in selected cases for vascular surgeons in the treatment of type II endoleaks after EVAR and TEVAR.

**Table:** 

Authors' roles & responsibilities	
FSLS ICG RSC PLSF	Substantial contributions to the conception or design of the work; or the acquisition, analysis, or interpretation of data for the work; final approval of the version to be published Substantial contributions to the conception or design of the work; or the acquisition, analysis, or interpretation of data for the work; final approval of the version to be published Substantial contributions to the conception or design of the work; or the acquisition, analysis, or interpretation of data for the work; final approval of the version to be published Substantial contributions to the conception or design of the work; or the acquisition, analysis, or interpretation of data for the work; final approval of the version to be published
